# Laser-Induced Koebner-Related Skin Reactions: A Clinical Overview

**DOI:** 10.3390/medicina60071177

**Published:** 2024-07-20

**Authors:** Emmanouil Karampinis, Konstantina-Eirini Georgopoulou, George Goudouras, Vicky Lianou, Elli Kampra, Angeliki Victoria Roussaki Schulze, Efterpi Zafiriou

**Affiliations:** 1 Department of Dermatology, Faculty of Medicine, School of Health Sciences, University General Hospital of Larissa, University of Thessaly, 41110 Larissa, Greece; ekarampinis@uth.gr (E.K.); geogkounto@hotmail.com (G.G.); lvicky89@hotmail.com (V.L.); roussaki@otenet.gr (A.V.R.S.); 2 Department of Dermatology, General Hospital of West Attica “Agia Varvara”, 12351 Athens, Greece; koneirgeo@gmail.com

**Keywords:** Koebner phenomenon, lasers, hair removal, psoriasis, vitiligo, port-wine stain, eczema, pathergy

## Abstract

The Koebner phenomenon (KP), also known as the isomorphic response, describes the process by which new lesions that are clinically and histologically identical to a patient’s existing skin disease develop following trauma. Many skin diseases exhibit this characteristic, with variations that include possible, questionable, and pseudo-Koebner reactions, with the latter category occurring due to infectious agents seeding at a trauma site. Laser application, a type of controlled skin injury used for improving cutaneous lesions and skin rejuvenation, is also considered a form of trauma. This raises the question of whether controlled thermal injury can be regarded as a type of mechanical trauma capable of producing Koebner-related reactions. We conducted a literature review of cases or studies to identify laser-induced dermatoses that correspond to Koebner-related or pathergy reaction categories. As a whole, we identified nine case reports on true KPs, two cases on possible KPs, seventeen cases on laser-induced questionable KPs comprising cases of vasculitis, eczema or Meyerson reactions, and eruptive squamous atypia cases (ESA) as well as two pseudo-Koebner cases involving wart occurrences at laser application sites. Laser-induced Koebner reactions highlight several aspects of the KP. Firstly, the type of mechanical damage influences disease promotion, as different lasers are associated with different KPs. For example, hair removal lasers are linked with true and questionable KPs such as vasculitis while resurfacing lasers were found to be more connected with ESA occurrence. Secondly, the laser target is significant, with vascular laser application for port-wine stains tending to result in eczematous reactions, while hair follicle destruction can frequently lead to true KPs. Thirdly, the number of sessions matters; true KPs and eruptive squamous atypia questionable KPs typically appear after one to two sessions, whereas eczematous reactions require more sessions (at least four). Additionally, skin phototype is crucial, with darker phototypes showing a higher KP frequency as laser treatment for hypertrichosis relies on melanin absorption in the hair bulge or bulb for follicle destruction, as chromophore competes with the abundant melanin in the epidermis. Further research with larger-scale studies into trauma-specific Koebner reactions is vital for refining treatment protocols, minimizing post-laser adverse effects, and improving dermatological care outcomes.

## 1. Introduction

### 1.1. Koebner Phenomenon Basic Principles

The Koebner phenomenon (KP), which is also known as the isomorphic response, describes the new lesions that appear that are clinically and histologically identical to the patient’s existing skin disease. The KP is thought to be an immune-mediated reaction, involving cytokines and autoantigens triggered by a cutaneous mechanical disturbance [1]. This non-specific inflammatory process triggers and finally gives place to the disease-specific reactions. For example, in the case of Koebner-induced psoriasis lesions, T-cell-mediated reactions promote keratinocyte proliferation. The KP is thought to be triggered by skin injury such as traumas, pressure application, and skin irritations, such as photosensitivity reactions and tattooing. The reaction follows the route of cutaneous injury while the severity of the response varies, ranging from no visible response or improvement in symptoms to widespread, disseminated lesions across the body [2]. True KP involves psoriasis, lichen planus, and vitiligo, while it has been most thoroughly studied in psoriasis. The time from injury to the appearance of psoriasis ranges from 10 to 20 days but can be as brief as 3 days or as long as 2 years, indicating that the sensitivity level for developing the Koebner response is a unique trait of the patient’s skin [3]. With every triggering factor, which is likely to cause located or systemic psoriasis flare, dermatosis exacerbation is bound to the exposome factors of the patient in question, such as age, gender, stress levels, diet type, and co-morbidities as well as skin parameters such as phototype [4]. Concerning the KP triggering factor, the trauma must result in both epidermal cell injury and dermal inflammation. For example, suctioning blisters do not lead to KP-induced psoriasis, likely due to the limited epidermal–dermal separation and inflammatory reaction that were not enough to trigger the phenomenon. On the contrary, blister roof removal causes a further rupture of the epidermis and initiates Koebner response, with secondary dermal events being necessary for a psoriatic lesion formation [2,3].

Given the terminology of the KP as the development of characteristic lesions that match the patient’s pre-existing skin disease, occurring at sites of recent skin injury or trauma, it is necessary to distinguish the Koebner reaction from other types of skin lesions or reactions such as allergic or infectious dermatoses. Pseudo-Koebner phenomenon (pseudo-KP) occurs when a skin infection, such as human papillomavirus (HPV) warts, molluscum contagiosum, Kaposi sarcoma [5], monkeypox [6], or impetigo, spreads to previously uninfected skin areas due to a traumatic event that breaches the skin barrier [1]. Opposed to true Koebner, the formation of new lesions is caused by the transfer of infectious material from an existing site to traumatized healthy skin and the development of skin infection lesions. In the pseudo-Koebner phenomenon, the trauma type as well as the exposome and skin properties of the patient seem to have a role as important as in the KP. Tattoo-associated viral infections are a well-known example of pseudo-Koebnerization, in which patients exhibit a typical course of viral skin infections due to viremia within a new tattoo [6].

The Koebner-related phenomena mentioned above are well established, but there is a need for extensive research to understand the pathophysiological link between trauma and the exacerbation of skin conditions. There are also less clearly defined terms, such as possible Koebner-induced lesions and questionable Koebner reactions. The former refers to dermatosis flares in traumatized skin that partially meet the criteria for the Koebner response, while the latter includes disorders noted in case reports that have an uncertain connection to trauma [1].

The pathergy phenomenon shares similarities with the Koebner phenomenon, as both involve the development of new skin lesions at the sites of trauma or injury. In contrast to Koebner, the pathergy lesions are typically distinct from the natural lesions of the disease. Pathergy reaction is a common characteristic and diagnostic criterion in Behcet syndrome. Pathergy phenomenon describes skin hyper-reactivity triggered by minor trauma, manifesting as non-specific pustules, papules, or the enlargement of existing wounds following minor injuries, including blunt trauma. Apart from Behcet syndrome, pathergy reaction is noticed in Sweet syndrome, pyoderma gangrenosum, and multiple keratoacanthomas (KAs) eruption [7]. Multiple KAs can be presented in the content of genetic syndromes [8] or in the form of eruptive squamous atypia (ESA). Also, there is controversy if ESA is a Koebner- or pathergy-related reaction with authors reporting, especially post-surgery, ESA as a questionable Koebner reaction and post-laser ESA as a pathergy reaction [9,10]. According to the field cancerization hypothesis, tumor development arises from an abnormal epithelial whose cells share early mutations that predispose them to malignancy. When trauma affects this area, squamous cell carcinomas and KA-like lesions can occur. This is maybe a case where the different type of traumas can result in different reactions in the content of the same disease [10].

### 1.2. Laser Applications and Laser-Induced Trauma as Potential Trigger of KP Reactions

Laser therapy traditionally involves the application of monochromatic light for treating various medical conditions. Additionally, laser applications play a significant role in cosmetic dermatology. According to the American Society for Aesthetic Plastic Surgeons, laser hair removal and laser skin resurfacing rank among the top five minimally invasive cosmetic procedures, with the former in third place and the latter in fourth [11]. The increasing demand for these cosmetic services has sparked a greater interest in investigating the foundations and background of aesthetic medical treatments.

Laser application is a type of controlled skin injury targeting skin lesion improvement as well as skin texture improvement and rejuvenation. Lasers have been effectively used to treat various vascular lesions such as port-wine stains (PWSs), with the pulsed dye laser (PDL) considered the preferred choice for most vascular lesions due to its superior clinical efficacy and low-risk profile. A large spot size (5 to 10 mm) also allows for the quick treatment of large lesions. Lasers can also be employed to remove excessive and cosmetically troublesome hair resulting from hypertrichosis or hirsutism. Effective devices for this purpose include long-pulsed ruby and alexandrite lasers, a diode (810 nm), millisecond Nd, and intense pulsed light (IPL) [12]. Fractional CO_2_ and erbium lasers have proven effective in diminishing and eliminating facial wrinkles, acne scars, and sun-damaged skin. The high-energy, pulsed, and scanned CO_2_ laser is widely regarded as the gold standard for facial rejuvenation. The principles of laser-induced application in the above-mentioned scenarios are energy absorption and tissue destruction by the thermally mediated injury of microscopic tissue targeting based on chromophore–wavelength correlations [13]. Examples include the thermal destruction of vessels in the case of PDL application in PWS targeting hemoglobin or hair melanin in the case of hair removal lasers [12].

Therefore, questions arise whether controlled thermal injury can be considered as a type of mechanical trauma of the skin adequate to produce Koebner-related reactions. The thermal damage caused by CO_2_ lasers to the skin initiates cellular immune responses, such as the aggregation of neutrophils and macrophages, pathogenic pathways triggered as the skin’s response to trauma [14,15]. Additionally, cutaneous oxidative stress without epidermal damage is observed in patients undergoing hair removal with lasers or IPL [16]. Laser-induced complications include superficial burns, erythema, and blister formation as well as hyper- or hypopigmentation often resulting from improper laser use, incorrect laser choice, or extensive trauma. Thus, laser-induced trauma appears capable of producing skin mechanical injury and inflammatory processes, which are necessary components of Koebner reactions [17]. Since lasers are common cosmetic procedures, it is important to consider their impact on patients with diseases associated with Koebner reactions, who often question whether laser application would affect the course of their disease.

While the KP has been extensively documented in association with various physical traumas and dermatological conditions, there is a paucity of research specifically investigating the role of laser treatments as a potential trigger for the KP. Existing studies have primarily focused on mechanical injuries, surgical interventions, and other forms of skin trauma as contributing factors. However, due to the increasing use of laser therapies in dermatology for both cosmetic and therapeutic purposes, there is a critical need to understand whether and how laser application might induce this process. This gap in the literature leaves dermatologists without comprehensive guidelines or predictive markers for KP occurrence post-laser treatment, potentially compromising patient care and treatment outcomes. Therefore, this review aims to compile and analyze instances of laser-induced Koebner-related reactions, offering a detailed characterization of these occurrences. Additionally, it seeks to establish clinical parameters that can guide practitioners in managing and anticipating such reactions in patients undergoing laser treatments.

## 2. Materials and Methods

We conducted a literature review to identify cases of laser-induced dermatoses that, as shown in Table 1, are related to the KP or pathergy skin reactions. Our search included PubMed articles published up to the end of April 2024, using keywords like “Post-laser” OR “Laser-induced” AND “Koebner”. Additionally, we incorporated all terms listed in the columns of Table 1 related to KP reactions.

We included case reports in which laser applications were used for cosmetic purposes (hair removal, PWS whitening, skin rejuvenation, or tattoo removal) and the KP was investigated. The included cases reported confirmed the histopathology diagnosis of the dermatosis in question. We excluded cases reporting the use of intense pulsed light or cases presenting patients with post-laser allergic reactions, contact dermatitis, urticaria, and/or post-laser classic complications such as burns or hyper/hypopigmentation lesions as these reactions are out of the “Koebner” box. Non-English language articles were excluded from the review. For Fitzpatrick skin-type characterization, we reported only the specific phototype provided by the authors, avoiding reliance on clinical images due to the potential subjectivity and error in visual assessments.

## 3. Results

Based on our research, we investigated the Koebner-related skin reactions due to laser. Concerning true KP reactions, we found two studies focusing on psoriasis flare, seven studies on vitiligo, and zero about lichen planus. Also, we found one study with mixed true, possible, and questionable Koebner reactions; however, in that study, there was not a disease-specific correlation between the reactions and as a result, further conclusions regarding laser-induced psoriasis, vitiligo, and lichen planus could not be drawn. Concerning other Koebner-related reactions, we found 2 possible KPs and 17 questionable KPs. Laser-induced questionable KPs included three cases of vasculitis, six cases with eczema or Meyerson reaction, and eight cases with eruptive squamous atypia. Finally, we found two cases of pseudo-Koebner including wart occurrence in the laser application sites.

### 3.1. Laser-Induced Lesions Based on True Koebner Phenomenon

Although it was anticipated that more studies would be found, only three studies relating to psoriasis induced by cosmetic-purpose lasers were detected, including a case report, a retrospective study presenting a patient with de novo psoriasis due to laser hair removal, and a cross-sectional study including psoriasis patients where no new lesions were noticed, indicating that laser-induced psoriasis is a rare phenomenon (Table 2). No Koebnerization has been reported with excimer and pulsed dye lasers, which are used in the treatment of psoriasis. Also, we found a retrospective study with mixed Koebner reaction diseases including post-hair removal with alexandrite and ND-YAG.

In the cross-sectional study included, 38 patients with plaque psoriasis who underwent laser therapy in different body parts for rejuvenation, actinic keratosis, or acne scarring improvement were reviewed. The devices tested were the UltraPulse CO_2_ (Coherent, Santa Clara, CA, USA), NaturaLase Erbium-YAG (Focus Medical, Bethel, CT, USA), and Portrait PSR3 (Rhytec, Waltham, MA, USA). Cosmetic laser, however, is contraindicated in the skin area where the laser target co-exists with active psoriasis. It is worth mentioning that no hair removal lasers or diode were included in the study with no Koebner observation. The phototype of the patients examined were mostly I or II type while Fitzpatrick III was only reported in two cases while IV was not included [18].

No correlation with age, gender, or phototype was indicated, as only one case of laser-induced psoriasis was identified. In this instance, the application of a diode laser for hair removal led to the formation of multiple discrete erythematous scaly papules and a few plaques on the anterior and posterior aspects of both legs with a positive Auspitz sign. The patient was started on methotrexate, 12.5 mg per week, along with coal tar and salicylic acid ointment twice a day, and after eight weeks, the lesions cleared. In the case of laser-induced psoriasis, the authors proposed a differential diagnosis of lichenoid and contact allergic dermatitis [19]. In the cross-sectional study, higher wavelengths and therefore deeper penetration were assessed (CO_2_ lasers have a wavelength of 10,600 nm, erbium laser has a wavelength of 2940 nm), while the only laser type where Koebnerization was observed was the diode laser (805 nm). This absence of psoriasis flare following resurfacing lasers might be explained by the precise and controlled energy delivery provided by lasers, which unlike diffuse and uncontrolled skin injury, do not lead to Koebnerization. The destruction of hair follicles and surrounding dermal structures by the diode laser, on the other hand, can create a more pronounced inflammatory response, causing psoriasis via the Koebner phenomenon [18].

In the retrospective study found, which included mixed true and questionable KPs, laser hair removal-related KP occurred in almost 30% of the patients studied, indicating that it is a rare complication but underreported in the medical bibliography. The KP was related with alexandrite laser in 30% mainly after one session and ND-YAG in 55.55% after two sessions. However, due to the lack of psoriasis-specific findings, a definitive conclusion cannot be drawn [20].

Lichen reactions can also be observed as a side effect of cosmetic laser application. Lichen planus belongs to the true KP categories. Other types of lichen forms such as lichen sclerosus were considered as a possible KP. Lichen planus was also reported as the least frequent KP reaction, which occurred post-laser, and was only reported with laser hair removal including alexandrite laser and ND-YAG [20].

Vitiligo was a reported skin manifestation caused by the Koebner phenomenon following multiple laser treatment types from hair removal to skin rejuvenation. According to a cross-sectional study based on 30 vitiligo patients, the face and the legs were the two most affected body parts while erythema at a percentage of 50% or general post-laser cutaneous side effects such as crusting and blistering occurred in post-laser vitiligo occurrence. Vitiligo experts would use lasers in vitiligo patients when the disease is stable without disease activity signs or less aggressive laser modalities [21] (Table 3).

We also identified five cases of laser-induced vitiligo attributed to the Koebner mechanism and one patient with hypertrichosis that despite laser hair removal, Koebner was not observed [3,4,5,6,7,8,9,10,11,12,13,14,15,16,17,18,19,20,21,22,23,24,25,26,27,28]. Blistering was observed prior to PDL application, which was finally replaced by hyperpigmentation. Out of the five patients, three appeared with de novo lesions and two had a personal history of vitiligo or halo nevus. Concerning Fitzpatrick skin phototype, two out of five patients were type IV. Treatment types included epilation for one case, port-wine stain removal for two cases, hair removal for one case, and melasma treatment for one case. The devices used were as follows: alexandrite laser 755 nm (1/5, 20%), Nd 1064 nm (1/5, 20%), PDL 585 nm (2/5, 40%), and ablative laser (1/5, 20%). Vitiligo was located on the face (1/5, 20%), legs (2/5, 40%), arms (1/5, 20%), shoulder (1/5, 20%), and chest (1/5, 20%). Vitiligo onset ranged from 2–28 weeks, with two cases not specifying the interval. Treatments for laser-/IPL-induced vitiligo included topical corticosteroids, topical calcineurin inhibitors, 308 nm excimer laser, and narrowband UVB.

In summary, we found six patient case reports that developed a true Koebner reaction following laser application (five vitiligo-induced [22,24,25,26,27], one psoriasis occurrence [19]). The above-mentioned findings make vitiligo the most frequently reported laser-induced dermatosis by the KP (62.5%). This finding is supported by the fact that the only populated study concerning laser-induced Koebner lesions was about vitiligo–leukoderma. However, in a retrospective study focusing on hair removal lesions, psoriasis as the Koebner phenomenon was reported in 41% of the patients who had a relevant dermatosis and undergone laser hair removal [20]. Three out of six case reports (50%) were de novo manifestations of a skin disease contrary to the concept of a Koebner-positive patient. The occurrence of a new skin complication unrelated to previous skin disease was reported in a low percentage (almost 2%) [20]. The majority of the patients included were female patients (6/6, 100%) due to the fact that they are more exposed to cosmetic procedures. The range of age limits of the patients was from 7 to 39 years old. Also, four out of six (66.6%) were attributed to hair removal applications, making it the most likely laser cosmetic procedure that can trigger the Koebner phenomenon. A total of 50% of the vitiligo-specific cases were also attributed to laser hair removal applications.

Additionally, all the patients with a reported phototype and known laser Koebner reaction had Fitzpatrick IV [19,23,24], a dark-skinned phototype indicating that higher melanin levels may trigger a Koebner reaction when laser is applied. Additionally, the number of sessions appears to be crucial in eliciting a true KP reaction. In most cases, one to two sessions are sufficient to induce a true KP reaction, whereas in cases where no such reaction occurs, up to six sessions may be conducted without triggering the KP. This suggests that if the KP is about to occur, it will likely do so during the initial laser sessions, and if it does not, further sessions are unlikely to provoke it. Data about the timing interval between the laser and Koebner reaction were scattered while the post-inflammatory signs were more often erythema, while less common blistering and crusting can occur and precede the skin’s signs of the disease [18,21,23,24,25].

### 3.2. Laser-Induced Possible Koebner Reactions

Two cases with dermatosis were included in possible Koebner reactions and they were all caused by hair removal lasers. The laser-promoted skin diseases were lichen sclerosus and reactive perforating collagenosis (Table 4).

Following hair removal using a long-pulsed 1064 nm Nd:YAG laser, the first of the two patients appeared with perifollicular atrophic depigmented macules on the perineum and pubis, the skin regions where the laser application took place. The diagnosis of lichen sclerosus was also confirmed by dermoscopy and histopathology. The above-mentioned patient was treated with tacrolimus monohydrate 0.1 ointment twice daily for three months and mometasone furoate cream usp 0.1 once daily for one month, leading to significant improvement [28]. Another skin Koebnerization case related to laser application was a case of reactive perforating collagenosis, which is a rare skin disease characterized by the transepidermal elimination of altered collagen through the epidermis. The patient initially appeared with erythema and after 5–7 days, a lesion appeared on the posterior helical rim. The histopathology diagnosis was compatible with reactive perforating collagenosis. The appearance of such a lesion was attributed to the KP phenomenon [29].

### 3.3. Laser-Induced Lesions Based on Questionable Koebner Phrnomenon

Questionable Koebner reactions include all disorders with a dubious association with trauma. This group represents the intricate relationship between skin trauma and the exacerbation or spread of certain dermatological conditions. New lesions following trauma in some patients, but not consistently across all patients with the condition can be observed, and therefore this variability makes the trauma–lesion relationship “questionable”. Questionable Koebner reactions include vasculitis, eruptive keratoacanthoma reactions as well as eczema occurrence in the form of the Meyerson phenomenon or eczema dermatitis across the skin where laser treatment was performed.

Regarding the vasculitis category, one case of urticarial vasculitis, one case of leukocytoclastic vasculitis, and one case with Behcet [30,31,32] fall within the spectrum of vasculitis and were classified as questionable Koebner reactions (Table 5). The first one occurred in the first 48 h following the nature of urticaria skin reactions and the second within a week. Both cases referred to leg hair epilation using diode lasers. The authors proposed that the etiology factor in the urticarial vasculitis case could be a delayed hypersensitivity reaction in a subset of predisposed allergic patients. An antigen from the disrupted hair follicle may be the triggering factor. This mechanism has been proposed for laser-induced urticaria lesions [31]. Also, in the case of Behcet, the pathergy reaction, a characteristic reaction of this vasculitis type, happened post-laser in a 25-year-old man with known Behcet syndrome. Pustules in the dorsal region, anterior trunk, and thighs were the pathergy skin reaction observed three days after laser hair removal application [32].

In the case of vasculitis, the occurrence was affected by the nature of vasculitis in the urticaria form happening sooner. Two cases reported the occurrence after application to the legs and one on the back, while all of them were concerning hair removal lasers. An explanation behind this could be that hair follicles are surrounded by a rich network of blood vessels. Targeting these follicles can affect the surrounding vasculature, potentially leading to vasculitis in susceptible individuals [30,31,32].

In the case of atopic dermatitis, the Meyerson phenomenon, an eczematous halo surrounding a pre-existing PWS and eczematous reactions were noticed (Table 6). In both the cases of D. Pavlović et al., KTP was used and were patients treated for port-wine strains without any history of atopic dermatitis that developed within a week after laser application. Patients typically present with scaling, itchy, and inflamed lesions, which tend to appear predominantly or most severely within the boundaries of the PWS. Treatment with topical corticosteroids is generally effective in eliminating the eczema in those cases [33]. In another case series, the eczematous reaction appeared within one month with more severe eczematous reactions such as pruritic and eczematous lesions with exudation. Topical corticosteroids were used in those cases as well [34]. Also, the study of Fonder M et al. reported another two more cases, according to which a scaly, itchy patch appeared during laser application within the lesion. These patients had no prior history of dermatitis [35].

In a retrospective study, 46% of a population of 500 children treated for PWS lightening developed dermatitis. They were 13 years old or younger, and all had a personal or family history of atopy. In 82% of the cases, dermatitis appeared after at least five sessions. Histopathologic examination showed spongiotic dermatitis. In all patients, the dermatitis resolved or improved with topical corticosteroid therapy [36].

The named Meyerson phenomenon or eczematous reaction was only reported after lightening procedures using pulsed dye lasers treating port-wine stains. A history of atopic dermatitis in most cases and in the large-scale study was associated with an eczematous reaction. The time of incidence varied from within weeks to months. Also, the age parameters also varied from infants to 17-year-old adolescents. The clinical presentation ranged from erythematous lesions within or borders to the port-wine lesion to crusted and oozing lesions with pruritus. Also, despite the incidence of the de novo appearance of true Koebner following laser application, eczematous lesions were induced after numerous laser applications (from 4 to 7 sessions). There were no significant differences in variations in spot size, radiant exposure/fluence, and pulse width that affect the laser’s interaction with the tissue and the potential for eczematous adverse effects [33,34,35].

Additionally, the correlation between eczema and the use of vascular lasers in treating port-wine stains (PWS) should be noted, as post-laser eczema or atopic flares have not been reported with other laser applications. The etiology proposed was tightly linked with the pathophysiology of PW and the already existing eczematous reactions that already exist within the lesion. It is suggested that this dermatitis might be caused by abnormal cytokine production, leading to inflammatory changes in the skin [36]. The vasodilatation and ectasia associated with PWS can affect this localized increase in proinflammatory mediators, which can either unmask an underlying atopic diathesis or exacerbate existing eczematous lesions. The additional inflammatory response induced by the PDL treatment can further exacerbate eczema. The combined effect of PDL-induced inflammation and the pre-existing inflammatory milieu in PWS can lead to the worsening of eczematous lesions [35,37].

Other questionable Koebner reactions included eruptive keratoacanthoma or ESA, which is used to describe an idiopathic condition involving the occasional development of atypical yet well-differentiated keratinocytes (also referred to as eruptive keratoacanthoma) (Table 7). ESA should be suspected, instead of a genetic syndrome, by the existence of an irritating trigger that could cause KA presentation and is often misdiagnosed as cancer and treated with excisional surgery, which in turn can further trigger KP.

In the case of ESA, the most lesions occurred from 7 to 14 days after laser application with the 10th day being the most frequent time noted and the longest period being 6 weeks [38,39,40,41,42,43,44]. Most of them were due to CO_2_ resurfacing while a Pico laser in the red color of the tattoo seemed to trigger the ESA initiation. The age of the patients was higher than the previous laser application cases tested. Also, in one case, the authors characterized ESA as a pathergy reaction. In this case, small papules and pustules took place before the appearance of the crateform lesions while on another, scaly lesions predisposed the appearance of keratoacanthomas [9]. The most cases included laser application to the face while two cases reported KAs appearing in the vermillion borders and two of them in the legs. Also, ESA seemed to appear more often in the form of multiple KAs rather than a single lesion [38].

The majority of ESA cases occurred following ablative skin resurfacing procedures (five of the eight cases). This type of resurfacing primarily damages the epidermal layer, causing trauma to keratinocytes and resulting in inflammation. This phenomenon aligns with the observation that epidermal trauma contributes to the development of eruptive KAs in some instances. Similarly, devices that employ fractional photothermolysis cause epidermal damage through a different mechanism, creating microscopic areas of thermal necrosis. Additionally, these findings highlight that a history of premalignant and malignant skin lesions can play role in KAs development, irrespective of trauma. In fact, many patients who developed laser-related KAs had previous skin neoplasms, often located at or near the site of the laser treatment. Managing KAs remains challenging due to the unpredictable behavior of these neoplasms [38].

### 3.4. Laser-Induced Lesions Based on Pseudo-Koebner Phenomenon

In the case of pseudo-Koebner reactions related to laser applications, we detected two case reports with the appearance of warts following face rejuvenation (Table 8). Only one session was needed to promote this infection. In one instance, the lesions appeared after two weeks, and there was no relevant history of verruca vulgaris.

The micro-injuries or trauma caused by lasers can provide an entry point for HPV, leading to the formation of new warts. This damage to the skin barrier may facilitate the spread and establishment of the virus. In the cases of wart reactivation, laser treatments can activate latent viral particles in the skin, resulting in new wart development. Additionally, beyond the pathophysiology linking warts and laser treatments, the potential for cross-contamination must be considered. If proper sterilization techniques are not followed, there is a risk of cross-contamination with HPV during the laser procedure. However, this explanation is less plausible since HPV strains have not been detected in laser fibers after treating infected skin lesions, even without sterilization, though the importance of proper sterilization remains undiminished [45].

**Table 8:** Presenting pseudo-Koebner reactions triggered by lasers. The infectious agents in both cases reported were HPV.

Study	Laser Type	Laser Application Parameters	Age/Sex	Fitz	Indication	Treatment Status/Relevant Medical History	Sessions	Koebner Reaction
[46]	Fractional CO_2_	7 W, 3 passes periorally, and 2 passes the rest of the face	78/F	NM	Face rejuvenation	NM	One	Verucca vulgaris
[47]	Fractional CO_2_	NM	27/F	II	Face resurfacing	NM	One	Multiple plane warts

## 4. Discussion

KP has proven to be a challenging subject due to its vague terminology and the extensive list of dermatoses potentially associated with it under the category of possible or questionable Koebner reactions. The inclusion of pathergy reactions further complicates this understanding. Pathogenesis, along with these reactions, involves a non-specific inflammatory response that links mechanical trauma with the pathophysiology of true Koebner reactions, such as psoriasis, lichen planus, or vitiligo [3]. Oxidative stress is also implicated in the Koebner reaction, as the imbalance between antioxidant mechanisms and reactive oxidative substances can occur after trauma, such as laser treatments [1,3]. Oxidative stress is a term widely associated with various skin conditions, ranging from inflammatory dermatoses [48] to skin cancers [49]. However, possible or questionable Koebner reactions appear to be closely connected with the underlying disease pathophysiology rather than being merely a non-specific inflammatory response that precedes the formation of skin lesions.

Laser-induced Koebner reactions are a rare post-laser observance but they can occur in patients with disease susceptibility and prove many aspects of the KP (Table 9). Firstly, that the type of mechanical damage plays a contributable role in promoting the disease as different types of lasers were associated with different KP reactions. For example, hair removal lasers are linked with true KP [19,21,22,25] and questionable KP reactions including the vasculitis category [30,31,32] while resurfacing lasers are more connected with ESA [39]. In the first case, hair removal lasers target the melanin in hair follicles, causing localized, intense heat and potential trauma to the skin and the surrounding blood vessels. This intervention can trigger an inflammatory response and, in predisposed individuals, lead to true KP reactions or vasculitis [1]. In the case of ESA, CO_2_ or erbium lasers primarily target the superficial layers of the skin to remove damaged or aged skin and promote skin renewal and collagen production by controlled epidermal damage [38]. This controlled damage to the epidermis, leading to a robust healing response, can result, according to the field cancerous hypothesis, in the development of atypical squamous cells as part of the skin’s regenerative process, potentially leading to eruptive squamous [38].

Secondly, the laser target is also significant as it is connected to specific reactions. For example, the application of PDLs to PWS is more connected to eczematous reactions while hair follicle destruction tends to true KP. As mentioned, laser-induced eczema dermatitis might result from abnormal cytokine production, leading to inflammatory changes in the skin. The vasodilatation associated with PWS can influence this localized increase in proinflammatory mediators, potentially unmasking an underlying atopic diathesis or exacerbating existing eczematous lesions [33,34,37]. While these treatments cause superficial vascular damage, leading to eczematous reactions due to skin barrier disruption and localized inflammation, laser hair removal treatments cause deeper penetration to destroy hair follicles, causing more significant trauma that can trigger true KP reactions.

Thirdly, the number of sessions also plays a significant role. For example, ESA questionable KPs seem to be expressed after one to two sessions while eczematous reactions need more sessions (at least four according to the cases reported). The initial sessions in true KPs are sufficient to trigger the underlying pathophysiological processes in predisposed individuals. This phenomenon is more likely explained by the fewer targets appearing in each subsequent session as fewer hair follicles, for example, need to be targeted in each lesion. As a result, the trauma and subsequent inflammatory response produced are less in each session and therefore not adequate to elicit a visible response due to cumulative exposure [19,21,22,25]. The opposite seems to occur in the case of PWS lightening induced by vascular lasers, as a gradual inflammation build-up seems to take place. Repeated laser exposure increases the production of proinflammatory mediators and cytokines, eventually leading to dermatitis. This build-up also seems to be facilitated by the localized nature of PWS as the same area is repeatedly exposed to the laser. This concentration of treatment in a single area leads to a build-up of inflammatory mediators and predisposes the skin site to a higher risk of dermatitis [33,34,37].

Another note-worthy observation is that the skin phototype is of great importance in KP reactions as darker phototypes are connected with a higher KP frequency [19,23,24,28]. Laser treatment for hypertrichosis depends on melanin absorption in the hair bulge or bulb to destroy follicles. However, treating individuals with darker skin tones faces challenges in targeting follicles effectively due to competition from melanin in the epidermis. Even with cooling devices, these patients often experience increased discomfort during laser hair removal procedures due to higher melanin levels in their skin and subsequent resulting inflammation. Generally, post-laser complications such as dyschromia occur more frequently in non-Caucasian populations and KP reactions are not excluded. Consequently, it is advisable to avoid higher laser fluences and instead opt for more conservative treatment parameters [50,51].

Data from the timing between the KP reaction and laser application seem to be scattered. For example, wart pseudo-Koebner after resurfacing occurred after 2 weeks [48], the longest ESA detected post-laser was 6 weeks [39], while a Behcet pathergy reaction happened after 3 days [33] and urticarial vasculitis 48 h following laser application [32]. Age, female, and laser parameters seem to have less of a contribution as no association was found. Concerning diagnosis, all the KP lesions following laser application had the same histopathological profile as the classic skin lesions. Regarding the correlation between histopathology and dermoscopy, the dermoscopy examination of a lesion could be a preferred non-invasive diagnostic approach [52]. Also, classic signs observed in the clinical investigation remain present such as the Auspitz sign in psoriasis [19]. Also, in Koebner-related reactions, no psoriasis subtype change was observed as other triggering factors have been reported to modify plaque to pustular psoriasis [53]. Apart from predisposing factors such as dark skin, no protecting factors such as extrinsic environmental factors or vitamin D status have been notified [54,55] or studied further.

Another important aspect that was reported by those studies was the concern about laser application on active lesions. Although PDL therapy effectively lightens port-wine stains and provides lasting resolution of associated dermatitis, it should not be applied to inflamed areas until the inflammation subsides to reduce the risk of adverse effects from the laser treatment [34]. A similar approach should be adopted in the case of active psoriasis and vitiligo lesions for fear of immediate exacerbation of the lesion [21]. Also, the appearance of de novo skin dermatosis even in low frequency is a pattern not following KP principles [3].

Therefore, clinicians should be vigilant about post-laser lesions on skin sites where laser applications could trigger KP-related dermatosis flares, especially in patients with a relevant medical history. Consequently, a patient’s medical history should be thoroughly assessed before undergoing any laser treatment and any active lesions should be avoided when the laser is applied. Patients should also be monitored closely, particularly when superficial erythema is present, as this can be apart from a minimal burn, the initial phase of the Koebner phenomenon that would evolve into lesions of a skin disease flare. Additionally, as the histopathology and clinical manifestations of post-laser KP are identical to those of the related dermatosis, a careful clinical examination as well as a dermoscopy evaluation should be performed on any lesion appearing after laser therapy.

While our literature review provides a comprehensive overview of the current state of knowledge on post-laser Koebner-related skin reactions, several limitations must be acknowledged, particularly regarding our reliance on case reports. Case reports typically involve single patients or a small number of cases, which limits the ability to generalize findings to larger populations. Without control groups, it is challenging to establish causal relationships or compare outcomes effectively. Selection and reporting bias should also be acknowledged. Conducting prospective studies or randomized controlled trials as well as multicenter studies with diverse populations would provide a more comprehensive understanding of the research question.

Laser-induced KP serves as a significant example highlighting the necessity for additional research into how specific types of skin trauma exacerbate dermatoses. This phenomenon underscores the complexities involved in skin responses to laser treatments, particularly in predisposed individuals where new lesions or conditions can develop at the sites of trauma. Investigating the mechanisms underlying laser-induced KP can provide insights into how various skin disorders are triggered or exacerbated by therapeutic interventions. Such research is crucial for refining treatment protocols, minimizing adverse effects, and improving outcomes in dermatological practice.

## 5. Conclusions

Laser-induced KP reactions have been observed across various dermatoses, categorized into true, possible, questionable, and pseudo-Koebner reactions. The characteristics of the laser such as laser type (vascular, resurfacing, hair removal, etc.), target area (PWS, hair, etc.), number of sessions as well as the patient’s skin attributes (phototype), and the underlying pathophysiology of the condition contribute collectively to the laser-induced KP occurrence. Further research into trauma-specific Koebner reactions is essential for refining treatment protocols, reducing adverse effects, and enhancing outcomes in dermatological care.

## Figures and Tables

**Table 1:** Dermatoses with Koebner-related skin reactions adopted by the study of Sanchez D.P et al. [1], adding pathergy reactions analyzed in Ergun et al.’s study [7] with modifications derived from Koebner reaction terminology. Kaposi was added to pseudo-Koebner reactions as human herpesvirus 8 (HHV-8) infection manifestations [5] while monkeypox was also an addition as a new skin entity with pseudo-Koebner characteristics [6]. Eruptive squamous atypia (ESA) was placed both in questionable and pathergy reactions due to medical bibliography disagreements [9,10].

Koebner-Related Reaction	Terminology	Examples
True Koebner	Development of characteristic lesions that match the patient’s pre-existing skin disease, occurring at sites of recent skin injury or trauma	Psoriasis Vitiligo Lichen planus
Possible Koebner	Development of characteristic lesions that partially meet the criteria for the Koebner response	Lichen sclerosus/atrophicus Darier disease Perforating folliculitis Hailey–Hailey syndrome Reactive perforating collagenosis Erythema multiforme Kyrle syndrome
Questionable Koebner	Development of characteristic lesions, which are based on case reports or the limited medical literature, exhibiting some of the criteria for the Koebner response	Vasculitis Lupus discoid erythematosus Atopic dermatitis or eczema Pityriasis rubra pilaris Lichen niditus/amyloidosis Surgical trauma-induced eruptive squamous atypia Urticaria pigmentosa Dermatitis herpetiform Bullous pemphigoid
Pseudo-Koebner	Formation of new lesions caused by the transfer of infectious material from an existing site to traumatized healthy skin	Kaposi sarcoma Monkeypox Verruca vulgaris Molluscum contagium
Pathergy	Development of lesions that are typically distinct from the natural lesions of the disease	Behcet syndrome Laser-induced eruptive squamous atypia Sweet syndrome Pyoderma gangrenosum

**Table 2:** Presenting the results of a cross-sectional study that focused on Koebner reaction in psoriasis patients through different laser types as well as one case report with a patient experiencing psoriasis flare following laser hair removal.

Study	Laser Type	Laser Application Parameters	Mean Age (Years Old)	Sex (Female: Male)	Indication	Treatment Status	Sessions	Koebner Phenomenon Description
[18]	CO_2_ UltraPulse	300 mJ energy/pulse, 60 W average power, computerized pattern generator adjusted to density of 5, size of 9, and pattern of 5	52.2	NM	Rhytides	2/5 under methotrexate 3/5 without systemic treatment	1	No Koebner observed
[18]	Erbium-YAG	4 Hz, 6 mm spot size, and 700 mJ	42.2	NM	Rhytides and acne scarring	2/5 under Alefacept 3/5 without systemic treatment	1	No Koebner observed
[18]	Portrait PSR3	2–4 mJ, 2–4 passes	42.9	NM	Rhytides and acne scarring	2/8 under Efalizumab 2/8 under Etanercept 1/8 methotrecate 1/8 combination of Etanercept and methotrexate 2/8 without systemic treatment	(6/8) 1 session (2/8) 2 sessions	No Koebner observed
Study	Laser Type	Laser Application Parameters	Age/Sex	Fitzpatrick	Indication	Treatment Status	Sessions	Koebner Phenomenon Description
[19]	Hair removal diode laser	22 joules and 100 ms (1st session) to 22 joules and 30 ms (3rd session)	39/F	IV	Hair legs	None	3	De novo psoriasis reaction

**Table 3:** Presenting the laser application characteristics (laser parameters, sessions) and patient traits (age, gender) in laser-induced vitiligo case reports.

Study	Laser Type	Laser Application Parameters	Age/Sex	Fitz	Indication	Treatment Status	Sessions	Koebner Reaction
[21]	NM	NM	NM	I (0.4%), II (15.7%), III (46.6%) IV (34.7%), V (2.2%), and VI (0.3%)	Hair removal (46.7%) Skin rejuvenation (16.7%) Pigmented lesion improvement (13.3%) Vascular lesion improvement (3.3%)	NM	NM	Leukoderma or vitiligo
[22]	Alexandrite LHR (755 nm)	NM	28/F	NM	Hair removal	Relevant medical history	Two (appeared both following 1st and 2nd session)	Vitiligo/depigmenation patches
[23]	Alexandrite LHR (755 nm)	Pulse duration of 3 ms	24/F	IV	Hair removal	Not mentioned	Six	No Koebner observed
[24]	Pulsed dye laser	585 nm, fluence of 6.0–7.0 J/cm^2^, spot size 5 mm	18/F	IV	Port-wine stain whitening	De novo	Three	Vitiligo/depigmenation patches
[25]	Nd:YAG laser	NM	41/F	NM	Hair removal	Relevant medical history	One	Vitiligo lesion
[26]	Pulsed dye laser	585 nm, other parameters NM	7/F	NM	Port-wine stain whitening	Relevant medical history	Three	Depigmenation in the borders of treatment areas
[27]	Ablative laser	NM	35/M	NM	Melasma improvement	De novo	One	Depigmentation lesions

**Table 4:** Presenting cases with post-laser skin reactions listed in the possible Koebner phenomenon category.

Study	Laser Type	Laser Application Parameters	Age/Sex	Fitz	Indication	Treatment Status/Relevant Medical History	Sessions	Koebner Reaction
[28]	Long-pulsed 1064 nm Nd:YAG laser.	NM	43/F	IV	Hair removal	None	Six	Lichen sclerosus
[29]	Long-pulse diode laser	From 30 joules/100 ms pulse duration to 30 joules/30 ms pulse duration	40/M	NM	Hair removal	De novo	Two	Reactive perforating collagenosis

**Table 5:** Presenting cases of laser-induced vasculitis.

Study	Laser Type	Laser Application Parameters	Age/Sex	Fitz	Indication	Treatment Status/Relevant Medical History	Sessions	Koebner Reaction
[31]	Diode laser	800 nm, with pulse width of 5–30 ms, a spot size of 9 × 9 mm, 430 pulses at a fluence energy of 24 J/cm^2^	36/F	NM	Hair removal—epilation	NM	Two	Urticarial vascultitis
[30]	Diode laser (alexandrite laser)	810 nm	44/F	NM	Hair removal—epilation	Systemic lupus erythematous	Eight	Leukocytoclastic vasculitis
Study	Laser Type	Laser Application Parameters	Age/sex	Fitz	Indication	Treatment Status/Relevant Medical History	Sessions	Pathergy Reaction
[32]	NM	NM	25/M	NM	Hair removal	Medical history of Behcet	One	Pustules in the dorsal region, anterior trunk, and thighs as Behcet flare

**Table 6:** Presenting cases of questionable Koebner in the form of eczematous reaction following vascular lasers to PWS.

Study	Laser Type	Laser Application Parameters	Age/Sex	Fitz	Indication	Treatment Status	Sessions	Koebner Reaction
[33]	KTP	532 nm with 3 and 4 mm spot sizes, radiant exposures 12 to 28 J/cm^2^, pulse widths 15 to 30 ms	12/F	NM	Port-wine stain improvement	Previously treated with ND-YAG (1054 NM)	NM	Meyerson phenomenon within the port-wine strain
[33]	KTP	532 nm with 4 mm spot size, 20 J/cm^2^ radiant exposure, 15 m	12/F	NM	Port-wine stain improvement	Previously treated with KTP (532 nm)	Four	Meyerson phenomenon within the port-wine strain remnants
[34]	VersaPulse	532 nm, had additional settings of a pulse width of 10–30 ms and a fl uence of 16–26 J/cm^2^.	17/M	NM	Port-wine stain improvement	Previously treated with VersaPulse (532 nm)	Seven	Eczematous dermatitis reaction
[34]	VersaPulse	532 nm with a pulse width of 10–30 ms and a fluence of 16–26 J/cm^2^	15/F	NM	Port-wine stain improvement	Previously treated with VersaPulse (532 nm)	Four	Eczematous dermatitis reaction
[35]	Cynosure flash lamp pulsed tuneable dye laser (585 nm)	585 nm, energy fluence 4.0–4.5 J/cm^2^, spot size (10 mm)	0/-	NM	Port-wine stain improvement	Previously treated with Cynosure (585 nm)	Five	Eczematous dermatitis reaction
[35]	Cynosure flash lamp pulsed tuneable dye laser (585 nm)	585 nm, energy fluence 3.8–4.5 J/cm^2^, spot size (10 mm).	1/-	NM	Port-wine stain improvement	Previously treated with Cynosure (585 nm)	Five	Eczematous dermatitis reaction

**Table 7:** Showing cases with post-laser eruptive squamous atypia (ESA).

Study	Laser Type	Laser Application Parameters	Age/Sex	Fitz	Indication	Treatment Status	Sessions	Koebner-Related Reaction
[38]	Combination full-face fractional CO_2_ along with full-field ablative perioral and periocular erbium laser resurfacing	NM	58/F	III	Resurfacing	NM	One	Eruptive squamous atypia
[39]	Fractional carbon dioxide (CO_2_) laser	NM	60/F	NM	Resurfacing	NM	One	Eruptive squamous atypia
[40]	Picosecond laser	532 nm, NM	33/M	NM	Tattoo removal	NM	Two	Eruptive squamous atypia
[9]	Fractional carbon dioxide (CO_2_) laser	NM	67/W	NM	Dermatoheliosis treatment	Nm	One	Eruptive squamous atypia
[41]	Erbium:YAG ablative laser	2940 nm	47/F	II	Face resurfacing	NM	One	Eruptive squamous atypia
[42]	Erbium-doped laser	1550 nm, energy 12 mJ, spot size 15 mm, wavelength	42/F	III	Photoageing actinic keratosis and lentigines	NM	Two	Eruptive squamous atypia
[42]	Erbium-doped laser	1550 nm, energy 50 mJ, spot size 15 mm.	63/F	III	Photoageing actinic keratosis and lentigines	NM	Two	Eruptive squamous atypia
[43]	Carbon dioxide (CO_2_) laser	0.12 mm spot size laser on the face at 15 mJ (density 2, 1 pass) and perioral treatment at 15 mJ (density 3, 1 pass).	68/F	NM	Resurfacing	History of NMSC	One	Eruptive squamous atypia

**Table 9:** Summarizing the predisposing factors for a post-laser KP reaction occurrence.

Predisposing Factors for Developing a Post-Laser KP-Related Skin Lesion
(1) Medical history of skin disease that presents with KP-related manifestations (such as psoriasis, lichen planus, vitiligo, etc.). (2)Type of laser mechanical trauma Hair removal lasers are linked with true and questionable KP reactions such as vasculitis while resurfacing lasers are more connected with eruptive squamous atypia. (3) Laser target Application of vascular lasers to PWS is more connected to eczematous reactions while hair follicle destruction tends to true KP reactions. (4) Number of laser sessions True and questionable KP reactions related to ESA typically appear after 1 to 2 sessions, whereas eczematous reactions generally require more sessions to develop, usually at least 4 according to reported cases. (5) Skin phototype Darker skin phototypes are associated with a higher frequency of KP.

## Data Availability

The data described in this study are available upon request from the corresponding author.
